# CD14^+^CD16^+^ monocyte-derived TREM2 macrophages promote lung fibrosis in systemic sclerosis–interstitial lung disease

**DOI:** 10.3389/fimmu.2026.1748574

**Published:** 2026-05-29

**Authors:** Jee Young Kim, Yong Jin Kim, Sang Jin Lee

**Affiliations:** 1 Cardiovascular Research Institute, Daegu, Republic of Korea; 2 Department of Pathology, School of Medicine, Kyungpook National University, School of Medicine, Daegu, Republic of Korea; 3 Bio-Medical Research Institute, Kyungpook National University, Daegu, Republic of Korea; 4 Division of Rheumatology, School of Medicine, Kyungpook National University, Daegu, Republic of Korea

**Keywords:** biomarkers, lung fibrosis, monocyte-derived macrophages, single-cell RNA-sequencing, systemic sclerosis–associated interstitial lung disease

## Abstract

**Background:**

Scar-associated macrophages (SAMs), defined by triggering receptor expressed on myeloid cells 2 (TREM2) and/or glycoprotein-NMB (GPNMB) expression, have been implicated in lung fibrosis. This study investigated the characteristics and functional roles of SAMs in lung tissues from patients with systemic sclerosis-interstitial lung disease (SSc-ILD) and healthy controls (HCs).

**Methods:**

SAM-related transcriptional profiles of lung tissues from patients with SSc-ILD and HCs were analyzed using single-cell RNA sequencing (scRNA-seq). Immunofluorescence staining of lung tissues was performed to detect CD68-positive SAMs expressing TREM2 or CCL2. Monocytes from patients with SSc-ILD and HCs were differentiated with granulocyte–macrophage colony-stimulating factor (GM-CSF) or M-CSF. The population of TREM2- and GPNMB-expressing cells was assessed by flow cytometry, and the levels of CCL2 and TGF-β1 in culture supernatants were measured in an ELISA. The fibrotic effects of macrophages on fibroblasts were examined in co-cultures. TREM2 inhibition was used to determine functional relevance.

**Results:**

scRNA-seq revealed the expansion of TREM2 macrophages highly expressing SAM-associated genes in the lower lung lobes of SSc-ILD patients. Immunofluorescence showed increased numbers of CD68^+^TREM2^+^ and CD68^+^CCL2^+^ macrophages in SSc-ILD lung tissues compared to HCs. In GM-CSF-, but not M-CSF-induced CD14^+^CD16^+^ monocyte-derived macrophages from patients with SSc-ILD, the upregulated expression of TREM2, GPNMB, CCL2, and TGF-β1 recapitulated the transcriptional features of SAM. When these macrophages were co-cultured with fibroblasts, the production of collagen, α-SMA and TGF-β was enhanced. By contrast, TREM2 inhibition significantly reduced these responses, as determined at the bulk RNA-seq and protein levels.

**Discussion:**

Our study identified a GM-CSF–TREM2–TGF-β1 axis driving monocytes-derived profibrotic macrophage–fibroblast crosstalk in SSc-ILD. It also showed that, in the lungs of SSc-ILD patients, CD14^+^CD16^+^ monocyte-derived SAMs drive fibrosis through a TREM2-mediated pathway, which may provide new therapeutic targets.

## 1 Introduction

Systemic sclerosis (SSc) is a chronic autoimmune disorder characterized by fibrosis of the skin and internal organs, including the lungs and heart, as well as vascular abnormalities (1–3). In SSc, fibrosis is primarily driven by activated fibroblasts (myofibroblasts). In this process, macrophages play a central role by interacting with extracellular matrix (ECM)-producing myofibroblasts and endothelial cells. Together, these cells establish a complex signaling network that includes the secretion of profibrotic mediators, cytokines and chemokines, thereby contributing to the progression of SSc and its association with interstitial lung disease (ILD) (1, 4–7). The lung contains at least two ontogenetically distinct populations of alveolar macrophages: tissue-resident alveolar macrophages (TR-AMs) and monocyte-derived alveolar macrophages (Mo-AMs) (8, 9). Mo-AMs express higher levels of profibrotic genes than TR-AMs, and the selective depletion of Mo-AMs, but not TR-AMs, reduces lung fibrosis in mice (8).

Granulocyte-macrophage colony-stimulating factor (GM-CSF) was initially identified as a regulator of myeloid cell proliferation, differentiation and activation during hematopoiesis. However, GM-CSF also serves as a key modulator of inflammation and tissue remodeling by stimulating the pro-inflammatory activities of macrophages, including enhanced antigen presentation, increased cytokine secretion and metabolic reprogramming (10). Additionally, GM-CSF-induced macrophages contribute to tissue remodeling by producing profibrotic factors such as transforming growth factor (TGF)-β1 and interleukin (IL)-6, which activate fibroblasts and facilitate ECM deposition (10, 11). A recent study found that GM-CSF preferentially drives the differentiation of alveolar macrophages from circulating monocytes (12). These alveolar macrophages secrete key mediators of fibrosis, which suggests that GM-CSF regulates a subset of fibrosis-associated macrophages, including those expressing the triggering receptor expressed on myeloid cells 2 (TREM2) (11–13).

TREM2 plays a crucial role in immune regulation and tissue homeostasis (13, 14). Structurally, it consists of an extracellular V-type immunoglobulin domain, a short stalk and a transmembrane region that interacts with DNAX-activation protein 12 (DAP12), an adaptor protein involved in intracellular signalling (15). The TREM2 signaling pathway has been implicated in the upregulation of profibrotic transcriptional genes in macrophages, including *secreted phosphoprotein 1* (*SPP1*), *glycoprotein non-metastatic melanoma B* (*GPNMB*), and *fatty* a*cid binding protein 5* (*FABP5*) (13, 16). TREM2 was first studied in neurodegenerative diseases, such as Alzheimer’s disease (17), and later its relevance has expanded to other pathological conditions, including fibrosis. High levels of TREM2 expression have also been detected in macrophage subsets associated with the tumor microenvironment, atherosclerosis and metabolic disorders such as steatosis (13, 18–20).

TREM2 is frequently expressed in scar-associated macrophages (SAMs), characterized by the high-level expression of profibrotic genes, such as *SPP1*, *GPNMB*, and *FABP5*. TREM2–SAMs have been identified across multiple fibrotic organs, including the lung, liver, and kidney (21–23), where they contribute to fibrosis by promoting myofibroblast activation in response to local inflammatory and environmental signals. The differentiation of these cells is orchestrated by GM-CSF, IL-17A and TGF-β1, key cytokines present in abundance in fibrotic microenvironments (21). TREM2 functions as a sensor of environmental cues and a regulator of macrophage survival, which enables TREM2–SAMs to exert pro-fibrotic effects through their sustained interaction with fibroblasts. Given its role as a functional driver of fibrosis, TREM2 has emerged as a promising therapeutic target for controlling pathological macrophage activity in fibrotic diseases (13, 14, 21).

Despite these advances, the specific role of TREM2 macrophages in SSc-ILD patients remains poorly defined. In particular, both the programming of TREM2 macrophages by GM-CSF signaling and the contribution of these cells to fibroblast activation and ECM accumulation in the fibrotic lung are unknown. In this study, TREM2–SAM populations were characterized from the lung tissues of SSc-ILD patients and healthy controls (HCs). Their differentiation and function were investigated in co-cultures of human lung fibroblasts and peripheral blood mononuclear cells (PBMCs) from SSc-ILD patients or HCs. Transcriptomic analysis, immunofluorescence imaging, flow cytometry, ELISA and co-culture assays were employed to elucidate a GM-CSF–TREM2–TGF-β1 axis underlying monocyte-derived macrophage–fibroblast interactions and driving profibrotic remodeling in SSc-ILD patients.

## 2 Materials and methods

### 2.1 Study design

The objectives of this study were to identify the macrophage subsets in the lung tissues of SSc-ILD patients that expand during fibrosis and compare the characteristics of macrophages associated with lung fibrosis in SSc-ILD patients with those of macrophages from HCs. Publicly available scRNA-seq data (PBMCs from SSc-ILD patients: GSE 195452 and HCs: GSE 249584, and lung tissues from SSc-ILD patients and HCs: GSE128169 and GSE159354) was analysed to examine SSc-ILD related gene expression, and immunofluorescence imaging was performed to assess levels of cytokine production across macrophage subsets in 15 lung tissues (10 SSc-ILD patients and 5 HCs). Flow cytometry and cell culture experiments were performed to assess the effects of GM-CSF on SAM expression using PBMCs from SSc-ILD patients (n=6), SSc patients without ILD (SSc w/o ILD; n=3) and HCs (n=6). Co-cultures of human lung fibroblasts and GM-CSF–induced monocyte-derived macrophage subsets were established to investigate the impact of these macrophages on fibroblasts and to evaluate the effects of TREM2 inhibition on fibrotic activation in cells derived from SSc-ILD patients and HCs. This study was approved by the Institutional Review Board (IRB) and Ethics Committee at Kyungpook National University Hospital (2023-07-024). Written informed consent was obtained from all participants.

### 2.2 Single-cell RNA-sequencing, cell clustering, cell type annotation, and pseudotime trajectory analysis

Quality control, filtering, clustering, annotation, differential expressed gene (DEG), and pseudotime trajectory analyses were conducted on lung tissues and PBMCs. The analysis was performed using the Seurat R package (version 4.4.1, https://satijalab.org/seurat/), a widely used tool for clustering merged scRNA-seq data matrices. To correct for technical variation across datasets (i.e., batch effects), we applied the FindIntegrationAnchors and Integrate Data functions with the first 30 principal components in Seurat software (24, 25). This anchor-based integration approach mitigates batch effects by identifying shared cellular states across datasets. Cells identified as potential doublets or of low quality, defined as those with more than 4000 genes or fewer than 200 genes, were excluded based on gene expression metrics. The data were normalized and integrated, followed by the identification of highly variable genes using the Seurat function “Find Variable Genes.” These variable genes were subsequently used for principal component analysis (PCA) to reduce data dimensionality. Further dimensionality reduction was performed using the uniform manifold approximation and projection (UMAP) algorithm, utilizing 20 principal components and neighborhood relationships, with a clustering resolution of 0.7. Gene expression patterns were visualized through dot plots, feature plots and violin plots, following the guidelines provided by the Seurat package.

Analysis of DEGs was conducted using volcano plots to identify specific marker genes differentiating SSc-ILD patients from HCs. Upregulated genes were defined by an average log_2_ fold change (FC) > 0.58 and an adjusted *p*-value < 0.05, while downregulated genes were defined by an average log_2_ FC < - 0.58 and an adjusted *p*-value < - 0.05. Pseudotime trajectory analyses of macrophages in the integrated lung tissue data were performed using the order cell_function and the plot_cell functions, tracing the transition from ficolin 1 (FCN1) macrophages to TREM2 macrophages. The top 100 genes with an adjusted *p*-value < 0.05 were classified into two patterns and visualized in a heatmap along the pseudotime trajectory.

### 2.3 Immunofluorescence imaging

Formalin-fixed paraffin-embedded lung tissue samples from ten SSc-ILD patients and five HCs were deparaffinized, hydrated and subjected to antigen retrieval by incubation in 10 mM sodium citrate buffer (EBS011-250, Enzynomics, South Korea) at 99 °C for 20 minutes. After blocking with bovine serum albumin (BSA), tissue sections were incubated overnight with primary antibodies against CD68 (clone: FA-11, catalog no. 14-0681-82, ThermoFisher Scientific, Waltham, MA, USA), TREM2 (clone: 237920, catalog no. MAB17291, R&D system Minneapolis, MN, USA), and chemokine (C-C motif) ligand 2 (CCL2, also known as monocyte chemoattractant protein-1[MCP-1], clone: 29H86L56, catalog no. 700489, ThermoFisher Scientific). Subsequently, the sections were incubated for 2 hours with either goat anti-mouse IgG (H+L) cross-adsorbed secondary antibody, alexa fluor™ 647 (A-21235), donkey anti-rat IgG (H+L) highly cross-adsorbed secondary antibody, alexa fluor™ 488 (A-11006) and goat anti-rabbit IgG (H+L) cross-adsorbed secondary antibody, alexa fluor™ 555 (A-21428) from ThermoFisher Scientific. The sections were then co-stained with 4′,6-diamidino-2-phenylindole (DAPI, 50 ng/mL, 62248; ThermoFisher Scientific) and counterstained using Ultra Cruz Mounting Medium before cover slipping. Images were acquired at 40× magnification using a fluorescence microscope (DMI3000 B; Leica, Germany). Quantitative analysis of CD68^+^TREM2^+^ or CD68^+^CCL2^+^ positive cells/DAPI in confocal images was performed using ImageJ software.

### 2.4 Monocyte subsets culture and flow cytometric analysis

Peripheral blood samples were collected from SSc-ILD patients, SSc w/o ILD patients and HCs at Kyungpook National University Hospital (Daegu, Republic of Korea) between August 2024 and January 2026. Consecutive eligible individuals attending the outpatient clinic during the study period were invited to participate, and all participants provided written informed consent. PBMCs were isolated from whole-blood samples from SSc-ILD patients, SSc w/o ILD patients and age- and sex-matched HCs using Ficoll-Paque Plus^®^ density-gradient centrifugation (catalog no. 17-1440-02; GE Healthcare, Chicago, IL, USA). Clinical characteristics of the SSc-ILD patients are summarized in **Supplementary Table 1**. The isolated PBMCs were stained with CD14 (Alexa Fluor^®^ 488 mouse anti-human, catalog no. 562689; BD Biosciences, Franklin Lakes, NJ, USA) and CD16 (APC mouse anti-human, catalog no. 561304; BD Biosciences). Peripheral blood monocyte subsets (CD14^+^CD16^-^ and CD14^+^CD16^+^) were sorted from the stained PBMCs using the BD FACS Aria™ III Cell Sorter (BD Bioscience). The isolated monocyte subsets (CD14^+^CD16^-^ and CD14^+^CD16^+^) were re-suspended in monocyte attachment medium (catalog no. C-28051, PromoCell, Heidelberg, Germany) and allowed to attach for 1.5 hours at 37 °C in a 5% CO_2_ incubator. After attachment, the medium was replaced with M1-macrophage generation medium XF (C-28055, PromoCell) according to the manufacturer’s instructions. The monocyte subsets were cultured for two weeks and treated with GM-CSF (50 ng/ml, PromoCell) or M-CSF (30 ng/ml, 216-MC-010, R&D Systems) to induce macrophage differentiation.

For flow cytometric analysis, monocyte-derived macrophages were permeabilized using the FOXP3/Transcription factor staining buffer set (catalog no. 00-5523-00; eBioscience, San Diego, CA, USA) according to the manufacturer’s instructions. The macrophages were labelled with antibodies against anti-human CD68 FITC (clone no. eBioY1/82A (Y1/82A), catalog no. 11-0689-42, ThermoFisher Scientific), anti-human TREM2 alexa fluor^®^ 647 (clone no. 237920, catalog no. FAB17291R-025, R&D system) and anti-human GPNMB PE (clone no. HOST5DS, catalog no. 12-9838-42, ThermoFisher Scientific). Data acquisition was performed using the BD FACS Aria™ III Cell Sorter and analyzed with FlowJo software (BD Biosciences).

### 2.5 Cytokine measurement in culture supernatants

ELISA was used to quantify levels of CCL2 (MCP-1; BMS281), TGF-β1 (BMS2065), IL-6 (EH2IL6), and IL-4 (BMS225-2) from Thermo Fisher Scientific, and CCL18 (DCL180B; R&D Systems) in cell culture supernatants. The optical densities of the samples were measured at 450 nm using a spectrophotometer (VersaMax, Molecular Devices, LLC, San Jose, CA, USA). CCL2, TGF-β1, IL-6, and IL-4 and CCL18 concentrations were determined by analyzing the results with SoftMax Pro software (Molecular Devices).

### 2.6 Macrophage co-cultures with human lung fibroblasts

Macrophages differentiated from monocyte subsets by GM-CSF from SSc-ILD patients were cultured for 7 days and subsequently co-cultured with commercially sourced human lung fibroblasts (ACBR 469, Cell Systems, Kirkland, WA, USA) for 5 days, with or without treatment with a TREM2 inhibitor (BLP-NR018, Alomone Labs, Jerusalem, Israel). During the co-culture, a mixture of M1-Macrophage Generation Medium XF and fibroblast medium (CSS-A101, Cell Systems) was used to support cell growth and interaction.

### 2.7 Bulk RNA sequencing and western blot analysis

Total RNA was library preparation and sequencing were performed by Macrogen Inc. (Seoul, South Korea). Differential expression analysis was conducted using the DESeq2 package in R, with genes exhibiting an adjusted *p*-value < 0.05 and FC > ± 2 considered statistically significant. Data visualization including volcano plots was conducted using R.

For protein expression analysis, total protein was extracted, and the concentration measured. Equal protein loading amounts (20 µg) were resolved using 8% sodium dodecyl sulfate-polyacrylamide gel electrophoresis (SDS-PAGE) and the proteins were transferred to nitrocellulose membranes. After blocking with 5% skim milk (catalog no. 90002-594; Radnor, PA, USA), anti-collagen type 1 (COL1A1, 91144S), anti-collagen type 3 (COL3A1, 30565S), α-smooth muscle actin (α -SMA, 14968S) from Cell Signaling Technology, Danvers, MA, USA and anti-collagen type 7 (COL7A1, MA5-41570, ThermoFisher Scientific) were incubated overnight at 4 °C with the relevant primary antibodies. Glyceraldehyde-3-phosphate dehydrogenase (GAPDH, 2118S, Cell Signaling Technology) served as the loading control. Horse radish peroxidase (HRP)-conjugated secondary antibodies were applied for 1.5 h at room temperature. The blot was washed three times in Tris-buffered saline (TBS, Biosesang, Yongin, South Korea) containing 0.1% Tween 20 (CAS number 9005-64-5; Merck Millipore). Protein bands were detected by enhanced chemiluminescence (Fusion FX; Vilber, Collégien, France).

### 2.8 Statistical analysis

Each experiment was performed with a minimum of four replicates. Data analysis was conducted using GraphPad Prism 8 software from GraphPad Software (San Diego, CA, USA). The error bars represent the mean ± standard error of the mean (SEM), and statistical significance was considered at a *p*-value < 0.05.

## 3 Results

### 3.1 Immune cell remodelling in SSc-ILD is characterized by an expansion of TREM2 macrophages

**Figure 1:**
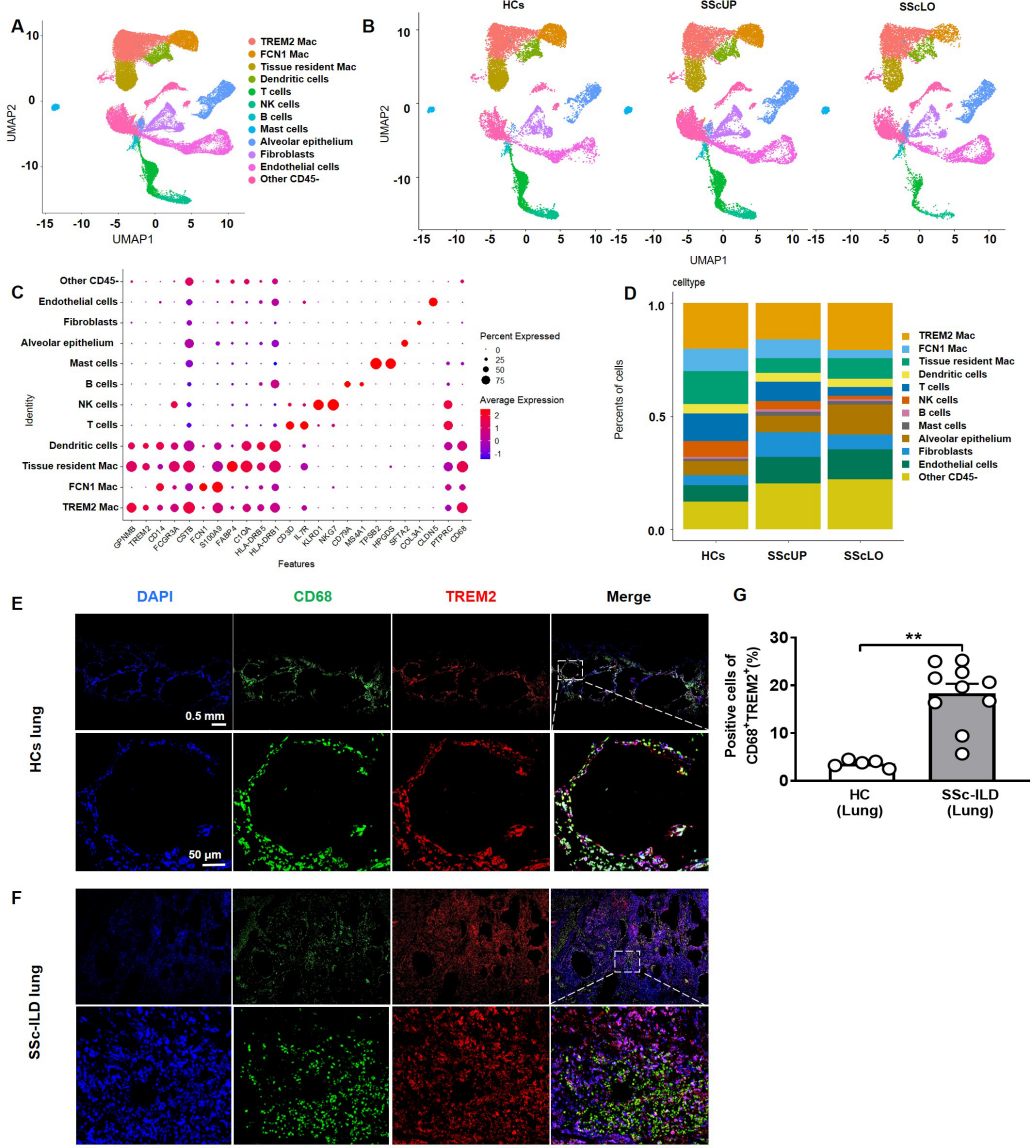
Increased TREM2 macrophages in lung tissue from SSc-ILD patients. **(A, B)** Uniform manifold approximation (UMAP) showing the distribution of cell types in lung tissues from patients with systemic sclerosis-associated interstitial lung disease (SSc-ILD) and healthy controls (HCs), with cell populations color-coded according to the legends. **(C)** Dot plot displaying representative marker genes for 12 identified cell types. **(D)** Bar graphs comparing cell type proportions between lung tissues from SSc-ILD patients and HCs. SScLO is characterized by an increased proportion of TREM2 macrophages with a decreased proportion of T and NK cells. **(E, F)** Representative low-magnification images showing CD68 (green) and TREM2 expression (red) in lung tissues from HCs (n = 5) and SSc-ILD patients (n = 10). DAPI (blue) stains nuclei. Merged images indicate co-localization of CD68^+^TREM2^+^ positive cells. Scale bar: 0.5 mm. High-magnification images correspond to specific regions selected from the low-magnification images, as indicated by the white dashed boxes, confirming the co-localization of CD68 with TREM2. Merged images highlight the presence of CD68^+^TREM2^+^ double-positive cells. Scale bar: 50 μm. **(G)** Quantification of CD68^+^TREM2^+^ positive cells/DAPI in lung tissues from SSc-ILD patients and HCs. Statistical analysis was conducted using an unpaired Student’s *t*-test, with n = 15. Statistical significance is indicated as **p < 0.01 SSc-ILD vs. HCs. Mac: macrophages.

The immune cell landscape in SSc-ILD patients was comprehensively characterized by analyzing publicly available scRNA-seq datasets from lung tissues (GSE128169) (26). SSc-ILD patients were stratified into two groups according to disease location, upper lobe (SScUP) and lower lobe (SScLO), and their immune cell profiles were compared with those of HCs. A uniform manifold approximation and projection (UMAP) analysis identified 12 distinct cell clusters, corresponding to eight immune and four non-immune cell populations, in lung tissues (**Figures 1A, B**). Immune cell subsets were obtained by isolating CD45^+^ cells using the pan-leukocyte marker, *PTPRC*. Immune cell types were classified based on canonical marker gene expression: TREM2 macrophages (*GPNMB*, *TREM2*, *FCGR3A* and *CSTB*), FCN1 macrophages (*FCN1* and *S100A9*), tissue-resident macrophages (*FABP4* and *C1QA*), dendritic cells (*HLA-DRB5* and *HLA-DRB1*), T cells (*CD3D* and *IL7R*), NK cells (*KLRD1* and *NKG7*), B cells (*CD79A* and *MS4A1*), and mast cells (*TPSB2* and *HPGDS*). Non-immune cell populations, including alveolar epithelium, fibroblasts, and endothelial cells, were annotated using *SFTA2*, *COL3A1* and *CLDN5*, respectively (**Figure 1C**). To confirm the robustness of the cell type classification, a heatmap of the top 20 DEG across clusters was generated (**Supplementary Figure 1**). Additionally, relative abundance of each cell population in SSc subgroups and HCs was quantified and visualized as stacked bar plots for total immune and non-immune cell populations (**Figure 1D**) and for CD45^+^ immune-cell subsets alone (**Supplementary Figure 2**). The SScLO subgroup showed a marked expansion of TREM2 macrophages together with a reduction in tissue-infiltrating T cells and NK cells, indicating a shift toward a myeloid-dominant immune landscape.

To validate *in situ* protein of TREM2 positive expression in SSc-ILD lung tissues, immunofluorescence staining for CD68 (green) and TREM2 (red) was performed on SSc-ILD lung tissues (**Figures 1E, F**). Quantitative analysis showed a significantly higher number of CD68^+^TREM2^+^ cells in the lung tissues of SSc-ILD patients than in those of HCs (**Figure 1G**), supporting a direct association between TREM2 and fibrotic progression.

For cross-validation of these expanded TREM2 macrophages, we analyzed an independent publicly available lung scRNA-seq dataset restricted to CD45^+^ immune cells (GSE159354) (27). Consistent with the results from GSE128169, SSc-ILD samples in this independent cohort showed an increased distribution and proportion of TREM2 macrophages compared with HCs. In addition, TREM2 macrophages in SSc-ILD exhibited higher expression of *SPP1*, *TREM2*, and *GPNMB* compared to HCs and exhibited higher expression of *SPP1*, *GPNMB*, *TREM2*, and *FABP5* compared with FCN1 macrophages (**Supplementary Figure 3**). Together, these findings support the reproducibility of our main observations across independent datasets.

### 3.2 SAM gene signatures are enriched in TREM2 macrophages from SScLO

**Figure 2:**
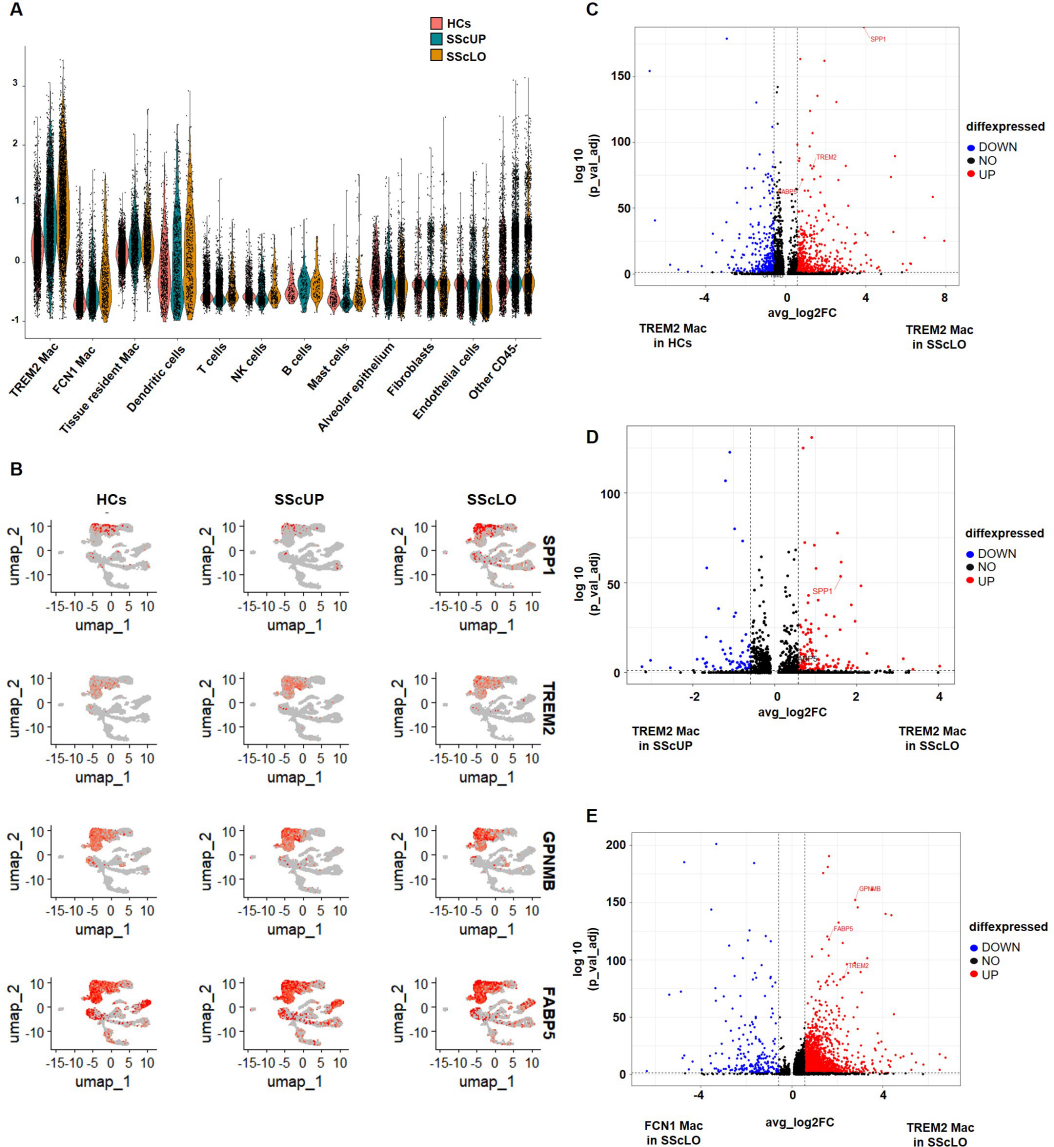
Elevated expression of scar-associated macrophage (SAM) genes in TREM2 macrophages from SScLO. **(A)** Violin plots showing the expression levels of SAM genes (*SPP1*, *TREM2*, *GPNMB* and *FABP5*) across cell types in different groups: HCs, SSc upper lobe (SScUP), and SScLO. The x-axis represents cell types, and the y-axis indicates the scoring of SAM genes expression. **(B)** Feature plots comparing the expression of the selected genes across different groups. The intensity of the red color represents the expression level of each gene. Cluster of TREM2 macrophages exhibited the highest expression of SAM-related genes compared to other immune cell types, and were most abundant in the SScLO group. **(C)** Volcano plot showing significantly upregulated genes (*SPP1*, *TREM2* and *FABP5*) in TREM2 macrophages from SScLO compared to HCs. **(D)** Comparison of TREM2 macrophages from SScLO and SScUP showing notable upregulation of *SPP1*. **(E)** Volcano plot comparing TREM2 macrophages with FCN1 macrophages from SScLO, revealing upregulation of *GPNMB*, *TREM2* and *FABP5*. Genes with significant upregulation are shown in red (average log_2_ fold change > 0.58 and adjusted *p*-value < 0.05), downregulated genes in blue (average log_2_ fold change < −0.58 and adjusted *p*-value < 0.05), and non-significant genes in black. Dotted lines indicate thresholds for statistical significance. diffexpressed: differential expressed.

SAMs are key drivers of fibrotic activation in lung fibrosis (21, 22). In this study, the expression of SAM-related genes was evaluated by calculating the module scores of four key markers (*SPP1, TREM2, GPNMB* and *FABP5*) using the AddModuleScore function in Seurat. GOBP_RESPONSE_TO_SAM module scores were visualized across cell subsets using violin plots to assess cell-type-specific enrichment (**Figure 2A**). Feature plots further indicated that the four marker genes were predominantly expressed in macrophages, with the highest enrichment in the SScLO subgroup, particularly within TREM2 macrophages (**Figure 2B**). These cells were the most abundant macrophage subset in this subgroup, and their expression of SAM-related markers was higher than in any of the other cell types. Together, these findings suggest an association in SScLO between the expansion of TREM2 macrophages, the enrichment of SAM-related genes, and fibrotic progression.

The expression of SAM genes was further explored in a DEG analysis comparing *SPP1*, *TREM2*, *GPNMB* and *FABP5* expression in TREM2 macrophages from SSc-ILD patients and HCs. In TREM2 macrophages, *TREM2*, *SPP1*, and *FABP5* expression levels were higher in SScLO than in HCs (**Figure 2C**). When TREM2 macrophages were compared between the SScUP and SScLO subgroups, only *SPP1* was significantly upregulated in SScLO (**Figure 2D**). In addition, within the SScLO subgroup, TREM2 macrophages showed higher expression of *SPP1*, *TREM2*, *GPNMB*, and *FABP5* genes than FCN1 macrophages (**Figure 2E**). Although *SPP1* expression was consistently upregulated in the SScLO subgroup in all comparisons, *TREM2* expression levels were comparable in the SScLO and SScUP subgroups. Previous studies showed that *SPP1* is significantly upregulated in alveolar macrophages from SSc-ILD patients, with the profibrotic transcriptional profile of these *SPP1*–expressing macrophages contributing to myeloid cell activation and disease progression (28, 29). Our findings of elevated *SPP1* expression in TREM2 macrophages within the SScLO subgroup are consistent with those reports and suggest a pivotal role for *SPP1* in mediating fibrotic responses in TREM2 macrophages of SSc-ILD patients.

### 3.3 The CCL2-CCR2 axis is activated in the lung macrophages and circulating monocytes of SSc-ILD patients

**Figure 3:**
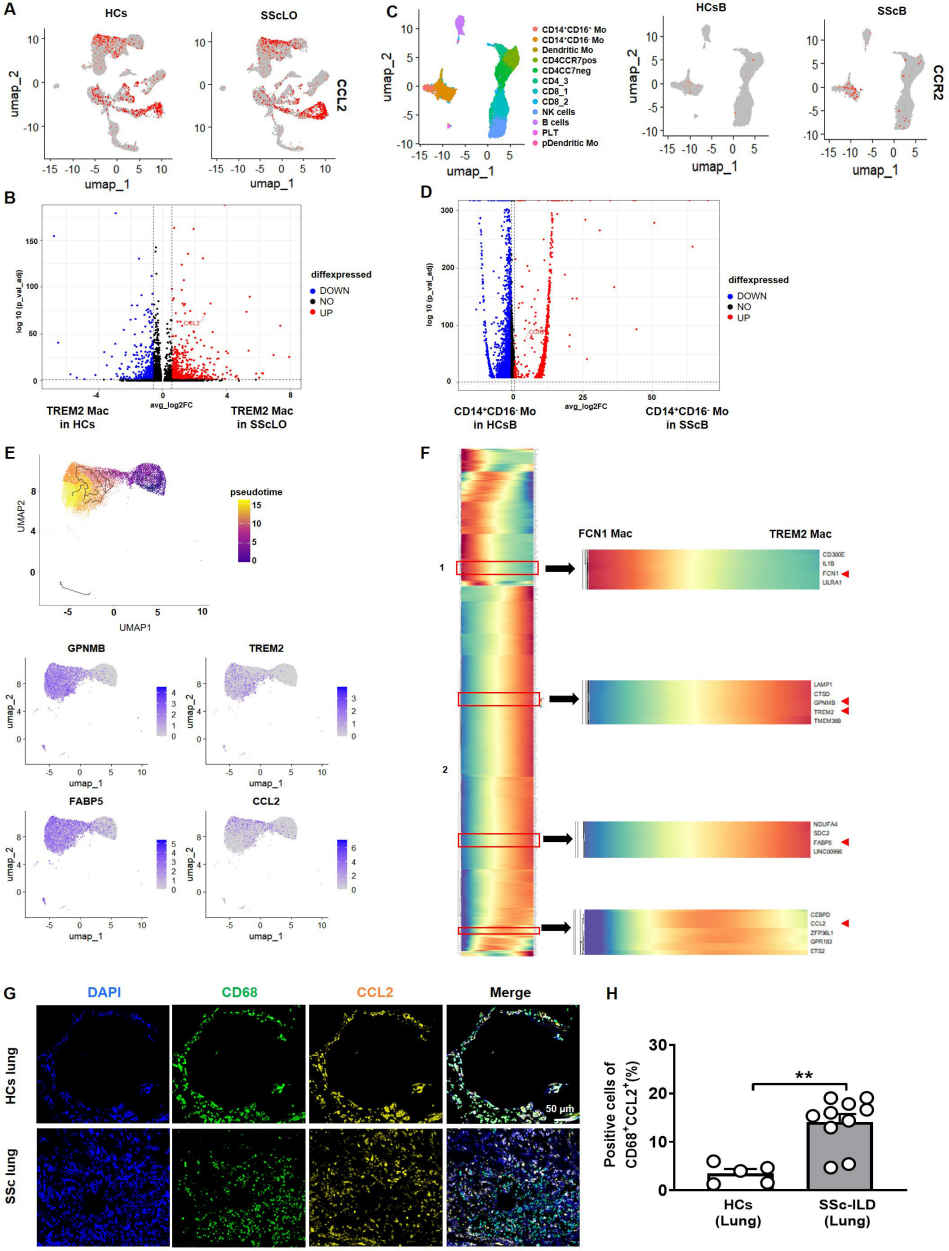
The CCL2–CCR2 axis associated with monocyte recruitment and CCL2 elevated in TREM2 macrophages from SSc-ILD. **(A)** Feature plots showing elevated *CCL2* expression in lung from SScLO compared to HCs. **(B)** Volcano plot showing increased *CCL2* expression in TREM2 macrophages from SScLO. **(C)** UMAP illustrating cell type distribution in PBMCs from SSc-ILD patients and HCs, with cell populations color-coded by type. Feature plot shows *CCR2* expression in PBMCs from both groups. **(D)** Volcano plot reveals significant upregulation of *CCR2* in CD14^+^CD16^-^ monocytes from SScB versus HCsB. Genes with significant upregulation are shown in red (average log_2_ fold change > 0.58 and adjusted *p*-value < 0.05), downregulated genes in blue (average log_2_ fold change < − 0.58 and adjusted *p*-value < 0.05), and non-significant genes in black. The dotted lines represent the thresholds for statistical significance. **(E)** Pseudotime trajectory of macrophages inferred from single-cell transcriptomic analysis. Cells are colored by pseudotime, progressing from purple (early) to yellow (late). Feature plots showing the spatial expression patterns of selected genes—*GPNMB*, *TREM2*, *FABP5*, and *CCL2*—that exhibit stage-specific expression along the trajectory. **(F)** Heatmap depicting gene expression dynamics across pseudotime, clustered into distinct modules. Zoomed-in heatmaps for each module highlight representative genes. These findings suggest that CCR2^+^ circulating monocytes may be recruited into the lung through the CCL2–CCR2 axis and subsequently differentiate along an FCN1-to-TREM2 trajectory toward a profibrotic macrophage state in SSc-ILD. **(G)** Representative images showing CD68 (green) and CCL2 expression (yellow) in lung tissues from HCs (n= 5) and SSc-ILD patients (n= 10). Nuclei are stained with DAPI (blue). Merged images indicate co-localization of CD68^+^CCL2^+^ double-positive cells. Scale bar: 50 μm. **(H)** Quantification of CD68^+^ CCL2^+^ positive cells in lung tissues from SSc-ILD patients and HCs. Statistical analysis was conducted using an unpaired Student’s *t*-test, with n = 15 (10 SSc-ILD patients and 5 HCs). Statistical significance is indicated as **p < 0.01 for SSc-ILD vs. HCs.

The chemokine CCL2 (also known as MCP-1) and its receptor CCR2 are critically involved in monocyte recruitment and macrophage accumulation (8). *CCL2* expression is elevated in the lung tissues of SSc-ILD patients, implicating *CCL2* in profibrotic macrophage differentiation and sustained inflammation (30, 31). A study in *Ccr2^-/-^* mice showed that the depletion of circulating monocytes ameliorates the severity of lung fibrosis (8). In this study, the subset – specific expression of *CCL2* and *CCR2* was assessed in feature plots and in a DEG analysis performed using scRNA-seq data from lung tissues and peripheral blood. *CCL2* expression was markedly higher in TREM2 macrophages from the lung tissues of the SScLO subgroup than in those from HCs (**Figures 3A, B**). *CCR2* expression in classical monocytes (CD14^+^CD16^-^) isolated from peripheral blood was significantly higher in SSc-ILD patients’ blood (SScB) than in HCs blood (HCsB) (**Figures 3C, D**). These findings suggest an enhanced *CCL2*–*CCR2* signaling in SSc-ILD that drives increased monocyte recruitment and infiltration into the fibrotic lung tissues of these patients. Alveolar macrophages are reconstituted during lung fibrosis through the differentiation of recruited Mo-AMs, which persist in the lung even after fibrosis resolution (8). To elucidate the differentiation trajectory of Mo-AMs in SScLO, a pseudotime analysis was performed to track the transition from FCN1 to TREM2 macrophages (**Figure 3E**). The pseudotime trajectory revealed two patterns of expression among the top 400 representative genes involved from the FCN1 to TREM2 macrophages transition: FCN1-associated genes (module 1) progressively downregulated but TREM2-associated genes (module 2-4) upregulated during the transition (**Figure 3F**). In cells positioned at the terminal end of this trajectory, corresponding to TREM2 macrophages, the elevated expression of *CCL2* in TREM2 macrophages suggested that monocyte**–**to**–**macrophage differentiation in SScLO culminates in the emergence of *CCL2*-producing macrophages, as shown in the feature plot (**Figure 3E**). In addition, *GPNMB*, *TREM2* and *FABP5* expression was higher in TREM2 than in FCN1 macrophages.

Given the pivotal role of CCL2 in recruiting CCR2^+^ monocytes and promoting fibrosis-associated tissue remodeling (13, 32), we next examined CCL2 expression in fibrotic lung lesions by immunofluorescence staining. Immunofluorescence images for CCL2 (**Figure 3G**), obtained from the same lung regions as in **Figures 1E, F**, showed a significant increase in CD68^+^CCL2^+^ macrophages in SSc-ILD. (**Figure 3H**).

Together, these findings suggest that enhanced CCL2 expression in fibrotic lung macrophages may contribute to CCR2 monocyte recruitment and macrophage accumulation within the fibrotic niche (33, 34). Based on the pseudotime trajectory, our results demonstrate that recruited monocytes progressively differentiate into TREM2 profibrotic macrophages within fibrotic lung tissues.

### 3.4 TREM2 and GPNMB expression and the production of CCL2 and TGF-β1 are enhanced in the GM-CSF–induced CD14^+^CD16^+^ monocyte-derived macrophages of SSc patients

**Figure 4:**
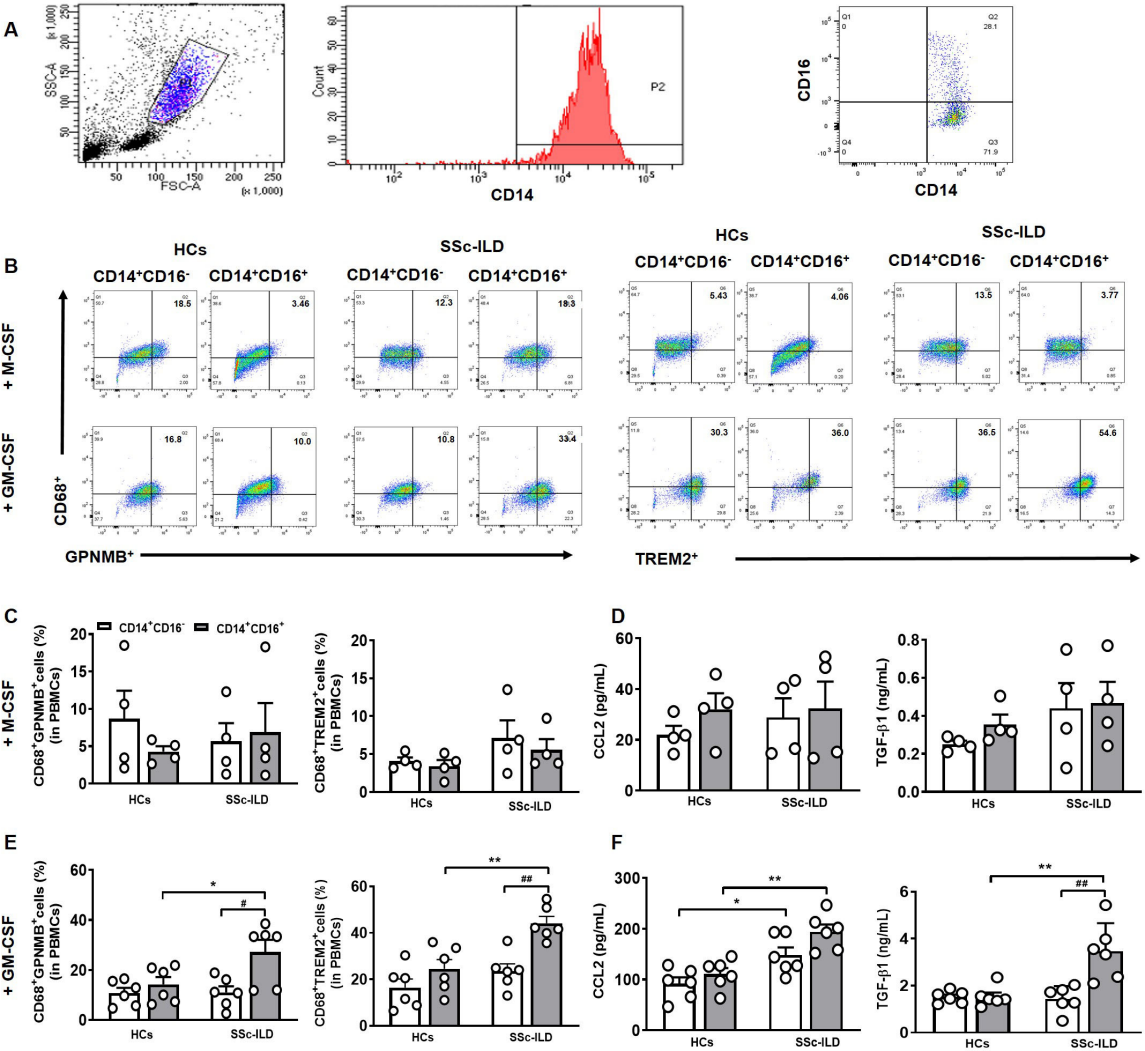
Granulocyte-macrophage colony-stimulating factor– (GM-CSF)–induced CD14^+^CD16^+^ monocytes from SSc-ILD patients showed an increased proportion of CD68^+^GPNMB^+^ and CD68^+^TREM2^+^ cells, along with elevated CCL2 and TGF-β1 levels in the culture supernatants. **(A)** Gating strategy for identifying CD14^+^CD16^-^ and CD14^+^CD16^+^ monocyte subsets. The first plot shows the selection of monocyte populations, the second shows CD14 expression, and the third shows CD14/CD16 subsets. **(B)** Representative flow cytometry plots showing the expression of GPNMB and TREM2 in M-CSF- or GM-CSF-induced macrophages derived from CD14^+^CD16^-^ or CD14^+^CD16^+^ monocytes from HCs and SSc-ILD patients. **(C, E)** Quantification of CD68^+^GPNMB^+^ and CD68^+^TREM2^+^ cells in macrophages treated with M-CSF or GM-CSF. A significant increase in both CD68^+^GPNMB^+^ and CD68^+^TREM2^+^ cells was observed in GM-CSF–induced CD14^+^CD16^+^ monocyte-derived macrophages from SSc-ILD patients compared to those from HCs, as well as in GM-CSF–induced CD14^+^CD16^-^ monocyte-derived macrophages from SSc-ILD patients. **(D, F)** CCL2 and TGF-β1 levels in culture supernatants measured by ELISA. A significant increase in both the levels of CCL2 and TGF-β1 were observed in GM-CSF–induced CD14^+^CD16^+^ monocyte-derived macrophages from SSc-ILD patients compared to those from HCs and to GM-CSF–induced CD14^+^CD16^-^ monocyte-derived macrophages from SSc-ILD patients. In contrast, in M-CSF–induced macrophages, no significant differences were observed in the proportions of CD68^+^GPNMB^+^ or CD68^+^TREM2^+^ cells, nor in CCL2 and TGF-β1 levels, between HCs and SSc-ILD patients. Statistical analysis was conducted using multivariate analysis of variance (MANOVA), followed by Tukey’s *post-hoc* multiple comparisons test. Data are presented as mean ± SEM, with n = 6 per group with GM-CSF treatment, n = 4 with M-CSF treatment. Statistical significance is indicated as *p < 0.05, **p < 0. for comparisons between SSc-ILD and HCs within the same subset; ^#^*p* < 0.05, ^##^*p* < 0.01 for comparisons between CD14^+^CD16^-^ and CD14^+^CD16^+^ subsets within SSc-ILD patients.

The scRNA-seq data indicated that TREM2 and FCN1 macrophages in SSc-ILD lungs are distinct myeloid subsets with divergent transcriptional profiles. Phenotypically, TREM2 macrophages show elevated *TREM2* and *FCGR3A* expression (**Figure 1C**) and correspond to CD14^+^CD16^+^ cells, encompassing both intermediate and non-classical monocytes. FCN1 macrophages are characterized by elevated *CD14* and *S100A9* expression (**Figure 1C**) and correspond to CD14^+^CD16^-^ classical monocytes. To model these subsets *ex vivo*, PBMCs were sorted into CD14^+^CD16^-^ (FCN1-like) and CD14^+^CD16^+^ (TREM2-like) populations and then stimulated with GM-CSF or M-CSF to evaluate their differentiation potential based on the expression of TREM2 and GPNMB, as key markers of SAMs (**Figures 4A, B**). GM-CSF significantly upregulated TREM2 and GPNMB expression in SSc-ILD derived CD14^+^CD16^+^ monocytes compared to CD14^+^CD16^+^ monocytes from HCs and CD14^+^CD16^-^ monocytes from SSc-ILD patients. CCL2 and TGF-β1 levels in culture supernatants were then measured by ELISA. CCL2 levels were significantly higher in GM-CSF-induced macrophages from SSc-ILD patients than in those from HCs, whereas TGF-β1 levels were markedly higher in CD14^+^CD16^+^ macrophages from SSc-ILD patients than in either CD14^+^CD16^+^ macrophages from HCs or CD14^+^CD16^-^ macrophages from SSc-ILD patients (**Figures 4C–F**). However, there were no significant differences in CCL2 and TGF-β1 expression among M-CSF-induced macrophages. Overall, our findings support that CCL2–CCR2-mediated monocyte recruitment and GM-CSF-associated macrophage activation may act together to promote SSc-ILD pathogenesis. CCL2–CCR2 signaling facilitates the recruitment of circulating CCR2 monocytes, and GM-CSF promotes their subsequent activation and differentiation into profibrotic CD14^+^CD16^+^/TREM2^+^ macrophages, ultimately amplifying tissue remodeling and fibrosis.

To evaluate whether GM-CSF–dependent changes were also observed in SSc w/o ILD patients, we performed parallel M-CSF– and GM-CSF–based differentiation experiments using CD14^+^CD16^-^ and CD14^+^CD16^+^ monocyte subsets from SSc w/o ILD patients. GM-CSF increased TREM2 and GPNMB expression and elevated CCL2 levels compared with M-CSF, whereas neither TGF-β1 levels nor the subset-dependent differences reached statistical significance (**Supplementary Figures 4 A–C**).

We additionally quantified macrophage-derived cytokines and chemokines associated with M1 like (IL-6) and M2 like (IL-4 and CCL18) polarization (35), in culture supernatants to determine whether GM-CSF–driven differentiation is accompanied by broader functional changes in the soluble mediator output beyond the TREM2/GPNMB–CCL2/TGF-β1 axis (36–38). Under GM-CSF conditions, IL-6 levels were elevated in both SSc-ILD and SSc w/o ILD compared with HCs. CCL18 levels were higher in SSc-ILD than in HCs, whereas IL-4 levels were comparable across all groups and subsets. Notably, in SSc-ILD, CCL18 levels differed between CD14^+^CD16^-^ and CD14^+^CD16^+^ subsets in a subset-dependent manner. In contrast, no significant differences were observed under M-CSF conditions (**Supplementary Figure 5**).

### 3.5 Profibrotic activation of GM-CSF–induced macrophages is dependent upon TREM2 expression

**Figure 5:**
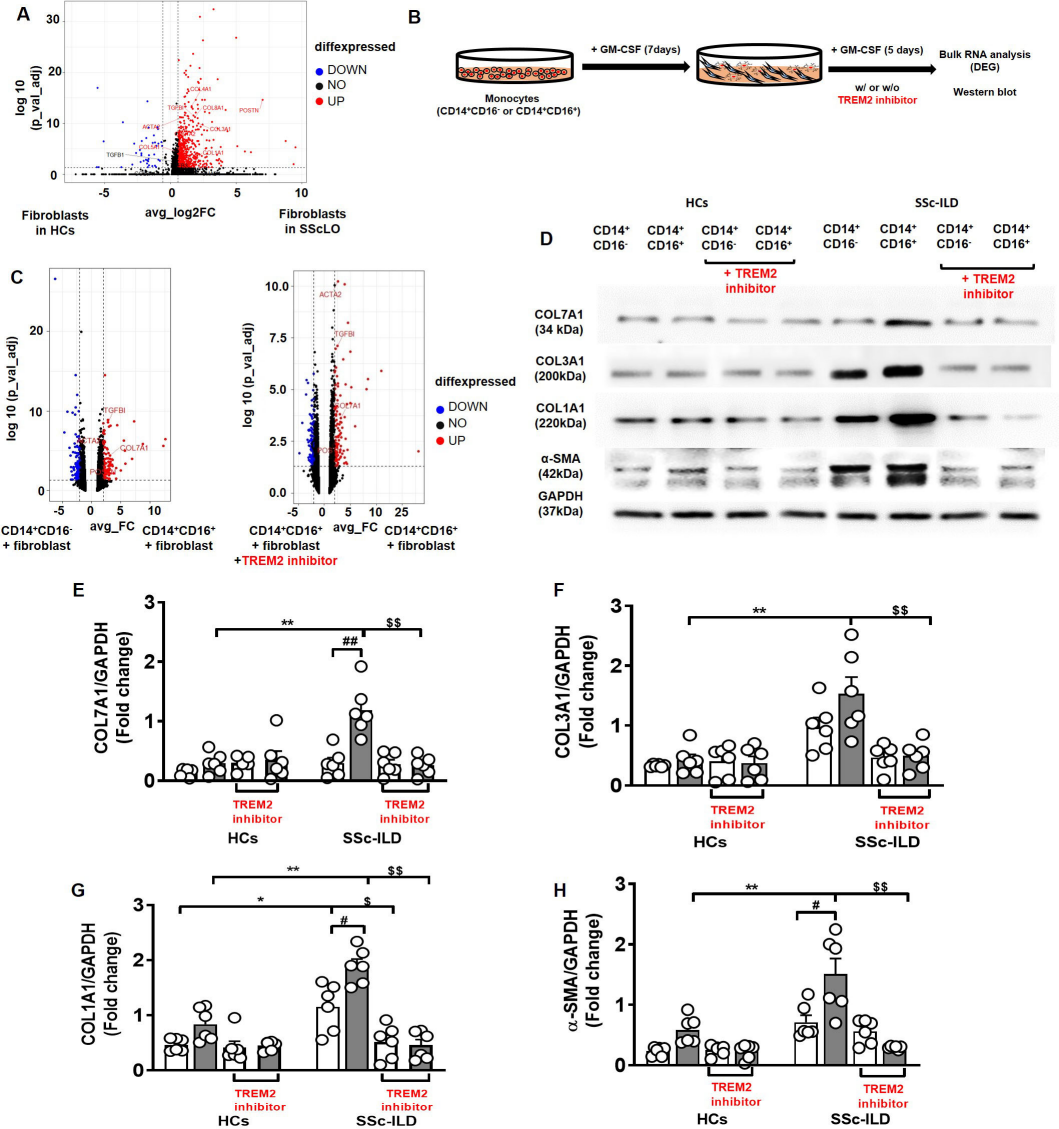
GM-CSF-induced CD14^+^CD16^+^ monocyte-derived macrophages promoted fibrotic activation of lung fibroblasts, an effect depends upon TREM2 expression. **(A)** Volcano plot showing DEG in fibroblasts from SScLO compared to those from HCs (GSE128169). Fibrotic activation genes, including *POSTN*, *COL1A1*, *COL3A1*, *COL4A1*, *COL5A1*, *COL8A1*, *TGFBI*, and *ACTA2*, were significantly upregulated in SScLO fibroblasts. **(B)** Schematic diagram of the co-culture experimental design. Monocytes (CD14^+^CD16^-^ or CD14^+^CD16^+^) were differentiated into macrophages using GM-CSF for 7 days, then co-cultured with human lung fibroblasts for 5 days with or without TREM2 inhibitor. Bulk RNA sequencing and western blot analyses were performed to assess gene expression and protein levels, respectively. **(C)** Volcano plots showing DEG in fibroblasts co-cultured with CD14^+^CD16^-^ vs. CD14^+^CD16^+^ macrophages (left) and CD14^+^CD16^+^ macrophages with or without TREM2 inhibitor (right). All macrophages were derived from SSc-ILD patients. Co-culture with CD14^+^CD16^+^ macrophages induced fibrotic gene expression (*POSTN*, *COL7A1*, *ACTA2*, and *TGFBI*), which was significantly reduced by TREM2 inhibition. Upregulated genes are shown in red (FC > 2, adj. p < 0.05), downregulated in blue (FC < -2, adj. p < 0.05), and non-significant genes in black. Dotted lines indicate statistical thresholds. **(D)** Representative western blot images of COL7A1 (34 kDa), COL3A1 (200 kDa), COL1A1 (220 kDa), and α-SMA (42 kDa); GAPDH (37 kDa) was used as a loading control. Conditions with TREM2 inhibitor are indicated in red. **(E–H)** Quantification of protein levels normalized to GAPDH: **(E)** COL7A1, **(F)** COL3A1, **(G)** COL1A1, and **(H)** α-SMA. Expression of COL7A1, COL1A1, and α-SMA was significantly elevated in fibroblasts co-cultured with CD14^+^CD16^+^ monocytes derived macrophages from SSc-ILD compared to HCs and CD14^+^CD16^-^ monocytes derived macrophages from SSc-ILD. COL3A1 expression was also elevated in SSc-ILD fibroblasts compared to HCs. TREM2 inhibition markedly reduced expression of all fibrotic markers. Statistical analysis was conducted using MANOVA, followed by Tukey’s *post-hoc* multiple comparisons test. Data are presented as mean ± SEM, with n = 6 per group. Statistical significance is indicated as *p < 0.05, **p < 0.01 for comparisons between SSc-ILD and HCs within the same subset; ^#^*p* < 0.05, ^##^*p* < 0.01 for comparisons between CD14^+^CD16^-^ and CD14^+^CD16^+^ subsets within SSc-ILD patients; ^$^*p* < 0.05, ^$$^*p* < 0.01 for comparisons between SSc-ILD and TREM2 inhibitor within the same subset.

The functional impact of GM-CSF–induced macrophages on profibrotic activation in SSc-ILD patients was investigated by co-culturing human lung fibroblasts with CD14^+^CD16^-^ or CD14^+^CD16^+^ monocyte-derived macrophages from HCs or from SSc-ILD patients, followed by bulk RNA sequencing and western blot analysis. Publicly available scRNA-seq data (**Figure 1B**) to compare fibroblasts from SScLO lungs with those from HC lungs revealed that the expression of the fibrosis-associated genes *COL1A1*, *COL3A1*, *COL4A1*, *COL5A1*, *POSTN*, *ACTA2* and *TGFBI* was significantly upregulated in SScLO fibroblasts (**Figure 5A**). We directly co-cultured GM-CSF–induced CD14^+^CD16^+^ or CD14^+^CD16^-^ monocyte-derived macrophages from SSc-ILD patients or HCs with primary human lung fibroblasts in the same well (**Figure 5B**). We then performed bulk RNA-seq and Western blot analyses on the resulting fibroblast–macrophage mixtures. Fibroblasts co-cultured with CD14^+^CD16^+^ macrophages from SSc-ILD patients exhibited markedly higher expression of fibrosis-associated genes, including *COL7A1*, *POSTN*, *ACTA2* and *TGFBI* compared to than those co-cultured with CD14^+^CD16^-^ macrophages (**Figures 5C**, left). Blocking TREM2 using anti-TREM2 peptides substantially reduced the expression of these genes (**Figures 5C**, right). Western blot analysis confirmed the increased expression of COL7A1, COL3A1, COL1A1, and α-SMA (encoded by *ACTA2*) in fibroblasts co-cultured with CD14^+^CD16^+^ macrophages from SSc-ILD patients compared to those co-cultured with either CD14^+^CD16^+^ macrophages from HCs or CD14^+^CD16^-^ macrophages from SSc-ILD patients. TREM2 inhibition significantly diminished collagen and α-SMA levels, supporting a role for TREM2 in mediating macrophage-induced fibroblast activation (**Figures 5E–H**). Similarly, ELISA analysis of culture supernatants showed that TREM2 inhibition reduced total TGF-β1 levels in cultures containing CD14^+^CD16^+^ macrophages from SSc-ILD patients. In contrast, although CCL2 levels were elevated in SSc-ILD patients compared with HCs across both CD14^+^CD16^-^ and CD14^+^CD16^+^ macrophage subsets, TREM2 inhibition reduced CCL2 levels, and this decrease did not reach statistical significance (**Supplementary Figure 6**). These findings demonstrate that profibrotic responses by TREM2 macrophages from the lung fibroblasts of SSc-ILD patients are amplified through TGF-β1 signaling. They also suggest a therapeutic strategy that, by targeting TREM2, attenuates the pathogenic macrophage-fibroblast crosstalk in SSc-ILD.

## 4 Discussion

**Figure 6:**
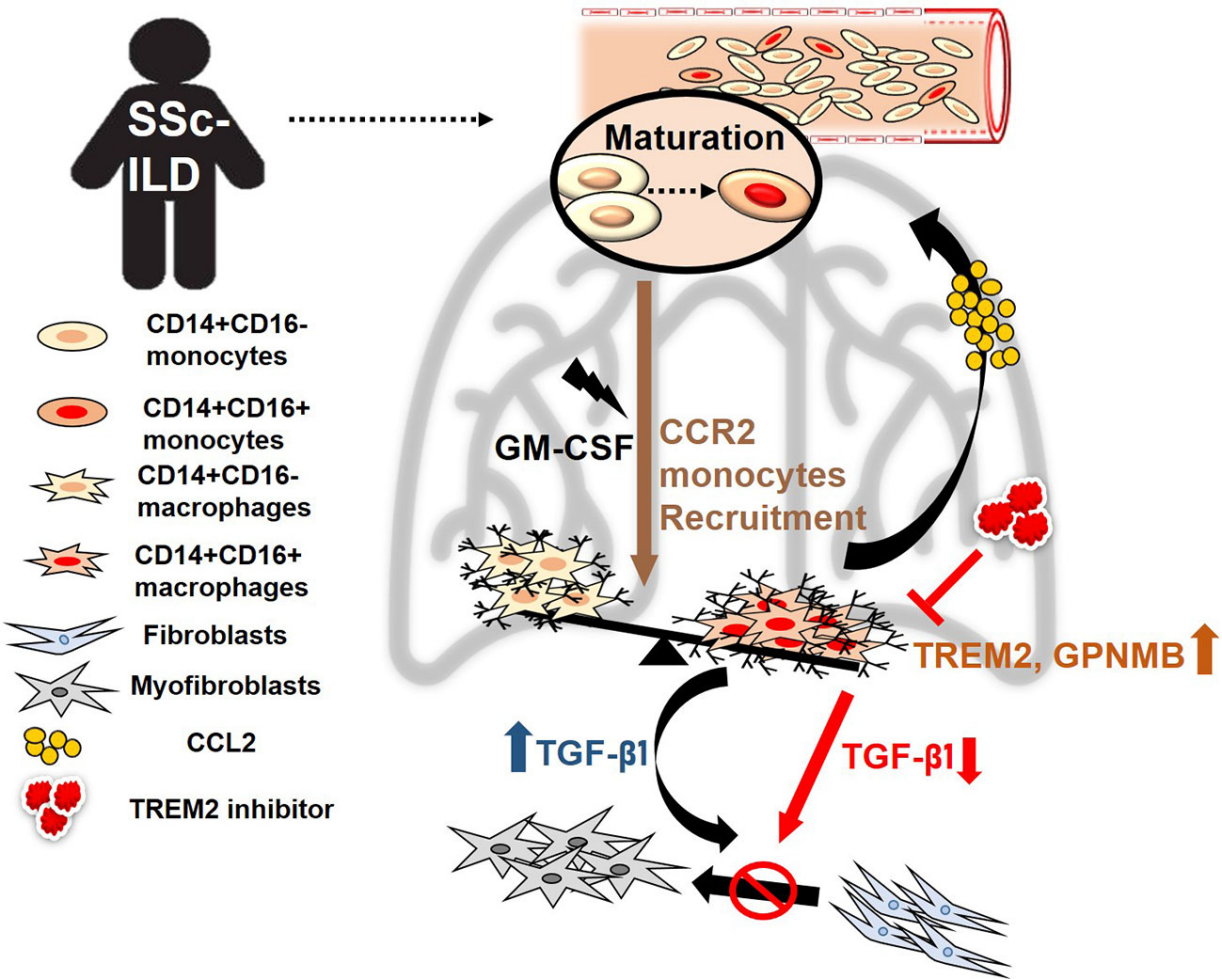
TREM2 macrophages and fibrotic progression in SSc-ILD. This schematic illustrates the proposed mechanism by which GM-CSF–induced monocyte-derived macrophages differentiate and promote lung fibrosis in patients with SSc-ILD. In SSc-ILD, recruited monocytes stimulated by GM-CSF upregulate *TREM2* and *GPNMB*, leading to increased secretion of cytokines such as CCL2 and TGF-β1. CCL2 promotes additional recruitment of monocytes via the CCL2-CCR2 axis. These TREM2^+^ macrophages enhance fibroblast activation through TGF-β1 production, driving fibroblast to myofibroblast differentiation and contributing to fibrotic remodeling in the lung.

This study investigated the role of TREM2 macrophages in the development of ILD from SSc patients. Using publicly available scRNA-seq data, flow cytometry, immunofluorescence, ELISA, and co-culture assays, we identified an expansion of TREM2 macrophages in the fibrotic lung tissues of these patients, particularly in the lower lobe. These TREM2 macrophages were characterized by the high-level expression of SAM-associated genes (*SPP1*, *GPNMB*, and *FABP5*) and enhanced CCL2 production, with the latter contributing to CD14^+^CD16^-^ classical monocyte recruitment via the CCL2–CCR2 axis. The results of our study suggest that, in SSc-ILD, GM-CSF preferentially drives the differentiation of CD14^+^CD16^+^ monocytes into TREM2 macrophages, with the subsequent secretion of elevated levels of CCL2 and TGF-β1. In our model, CD14^+^CD16^-^ classical monocytes with CCR2 expression in the circulation serve as a recruitment-competent precursor population mobilized to the lungs via the CCL2-CCR2 signaling axis. Upon infiltrating the fibrotic lung microenvironment, these recruited cells undergo *in situ* transcriptional and phenotypic reprogramming, maturing into CD14^+^CD16^+^ TREM2-high profibrotic macrophages. In co-culture experiments, TREM2 macrophages strongly induced fibrotic gene expression in lung fibroblasts, an effect dependent on TREM2. The inhibition of TREM2 significantly reduced fibroblast activation and the production of profibrotic cytokines such as TGF-β1. Together, these findings point to a GM-CSF–TREM2–TGF-β1 axis that mediates crosstalk between recruited monocytes and fibroblasts in SSc-ILD patients, and the potential of TREM2 as a promising therapeutic target (**Figure 6**).

Recent single-cell transcriptomic studies identified a population of monocyte-derived macrophages, termed SAMs, with conserved profiles across multiple fibrotic organs, including the lung, liver, kidney, and heart. SAMs are characterized by the enrichment of several genes, such as *TREM2*, *SPP1*, *GPNMB* and *FABP5*, and are functionally distinct from tissue-resident macrophages (13, 21, 26). SAM differentiation is regulated by GM-CSF and TGF-β1 (13, 21). Consistent with these findings, our investigation demonstrated the SAM-like transcriptional profile and behavior of CD14^+^CD16^+^ monocytes, but not CD14^+^CD16^-^ monocytes from SSc-ILD patients, including the elevated expression of *TREM2*, *GPNMB* and *CCL2*. Notably, TREM2 macrophages were specifically induced from recruited CD14^+^CD16^+^ monocytes by GM-CSF but not M-CSF induction. This observation aligns with previous reports showing that GM-CSF (but not M-CSF)-induced macrophages exhibit the phenotypic and functional traits associated with a profibrotic activation state (10, 39).

The cytokine/chemokine profiling highlighted phenotype-associated differences in macrophage-linked mediator programs across SSc groups in a subset-specific manner. Notably, under GM-CSF conditions, CCL18 was elevated in SSc-ILD compared to HC. This increase was confined to CD14^+^CD16^+^ derived macrophages, whereas CD14^+^CD16^-^ derived macrophages showed no significant difference from HC. This pattern is consistent with a previous report (38) showing that high serum levels of CCL18 are associated with SSc-ILD severity and disease progression, and it supports a macrophage-associated pro-fibrotic milieu preferentially enriched in patients with SSc-ILD. IL-6 levels were increased in both SSc-ILD and SSc w/o ILD compared with HC, aligning with evidence linking IL-6–associated inflammatory programs to SSc pathobiology and progression (36).

The immunofluorescence images revealed the significant co-localization of CD68^+^CCL2^+^ and CD68^+^TREM2^+^ macrophages in lung tissues from SSc-ILD patients. Furthermore, in co-cultures of lung fibroblasts and GM-CSF-induced CD14^+^CD16^+^ monocyte-derived macrophages from SSc-ILD patients, fibroblast activation was promoted by TREM2 and increased levels of the inflammatory cytokines CCL2 and TGF-β1 were secreted. These findings are consistent with the results of the scRNA-seq and pseudotime trajectory analysis, which showed the expansion of TREM2 macrophages and enhanced fibroblast activation in the lung tissues of SSc-ILD patients.

The pseudotime trajectory in this study also revealed a transition from FCN1 to TREM2 macrophages in populations recruited from circulating monocytes and an expansion of TREM2 macrophages in SScLO. These changes may reflect the extent of monocyte-to-macrophage maturation toward TREM2 macrophages driven by microenvironmental cues in the lung tissues of SSc-ILD patients. Classical CD14^+^CD16^-^ monocytes, which express CCR2, migrate to sites of inflammation, guided by the CCL2 gradients established by CCR2 (40–43). After infiltrating the lung tissue, these recruited cells differentiate toward an intermediate and non-classical-like phenotype, thereby acquiring CD14^+^CD16^+^ expression (43). This maturation process, potentially driven by GM-CSF induction, facilitates the transition of circulating classical CD14^+^CD16^-^ monocytes to non-classical CD14^+^CD16^+^ monocytes and, ultimately, into tissue-resident CD14^+^CD16^+^ macrophages. These cells subsequently contribute to the fibrotic process in SSc-ILD patients through TREM2-mediated profibrotic activities. This progression likely represents a spatiotemporal continuum of monocyte–to–macrophage differentiation, culminating in the accumulation of TREM2^+^ macrophages within fibrotic lung tissues in SSc-ILD patients.

TREM2, a cell transmembrane immunoglobulin-type receptor, regulates cellular functions through its transmembrane signaling adaptor DAP12 (13, 16). It is primarily expressed in immune cells, including dendritic cells, microglia, macrophages, and osteoclast precursors. TREM2 has been implicated in a wide range of cellular functions associated with neurodegenerative diseases, such as microglial proliferation, survival, phagocytosis, and inflammatory signaling, but also in regulating host responses to bacterial or viral infections (13, 17), anti-inflammatory responses, profibrotic behavior and macrophage survival (16, 17). As multi-tyrosine kinase inhibitor, approved for the treatment of lung fibrosis, nintedanib exerts its anti-fibrotic effect by targeting early events in TGF-β1 signaling and suppressing ECM production (44, 45). Similarly, in our study, TREM2 inhibition significantly reduced TGF-β1 production and the expression of profibrotic proteins in co-cultures of fibroblasts and GM-CSF-induced CD14^+^CD16^+^ monocytes from SSc-ILD patients.

Nevertheless, the limitations of this study should be taken into account. Because TGF-β1 was measured in macrophage–fibroblast co-culture supernatants, we could not fully distinguish macrophage-derived TGF-β1 from fibroblast-derived autocrine TGF-β1. Therefore, the observed reduction in total TGF-β1 following TREM2 inhibition should not be interpreted as suppression of macrophage-derived TGF-β1 alone. SSc-ILD is an intractable autoimmune disease characterized by progressive fibrosis of the skin and lung. Although serum autoantibodies, such as anti-centromere and anti-Scl-70 antibodies, are readily detectable and, are clinically associated with pulmonary fibrosis, and may trigger CD14^+^CD16^+^–TREM2 macrophages to participate the chronic inflammation, the precise functional pathogenesis of the autoantibodies on SSc-ILD is still unknown (2, 3).

Key findings of this study were validated using *in vitro* and *ex vivo* approaches. Therefore, our mechanistic and functional analyses of monocyte-derived macrophage subsets which were primarily conducted *in vitro*, do not directly establish their *in vivo* relationship during the development of SSc-ILD. Validation using an *in vivo* pulmonary fibrosis model, such as a bleomycin-induced murine model, and large-scale clinical study using molecular explorations for SSc-ILD patient would further strengthen the proposed profibrotic trajectory and its translational relevance. Particularly, *in vivo* functional studies targeting TREM2, are needed to determine how TREM2 macrophages play a causal role in SSc-ILD progression.

## 5 Conclusion

This study provides compelling evidence of a pivotal role for TREM2 macrophages in initiating and sustaining lung fibrosis in SSc-ILD patients. Its results suggest that TREM2 macrophages establish a profibrotic niche through TGF-β1 production and monocyte recruitment via the CCL2–CCR2 axis, thereby promoting fibroblast activation, ECM deposition and the exacerbation of inflammation. The demonstration of a mechanistic GM-CSF–TREM2–TGF-β1 axis of progressive fibrosis linking innate immune dysregulation to fibrotic remodeling points to this pathway as a promising target in fibrotic lung disease.

## Data Availability

The datasets presented in this study can be found in online repositories. The names of the repository/repositories and accession number(s) can be found in the article/**Supplementary Material**.
