# Supplementary material for: CD14+CD16+ monocyte-derived TREM2 macrophages promote lung fibrosis in systemic sclerosis–interstitial lung disease
Source: Front Immunol. 2026 May 29;17:1748574. doi: 10.3389/fimmu.2026.1748574 (PMC13260380; doi:10.3389/fimmu.2026.1748574)
Supplement: Supplementary file 1 [file DataSheet1.docx]

**Supplementary information**

**CD14^+^CD16^+^ monocyte-derived TREM2 macrophages promote lung fibrosis in systemic sclerosis–interstitial lung disease**

Jee Young Kim^1^, Yong Jin Kim^3,4^ and Sang Jin Lee^1,2,4^

^1^Cardiovascular Research Institute, ^2^Division of Rheumatology, Department of Internal Medicine,

^3^Department of Pathology, School of Medicine, Kyungpook National University, School of Medicine, Daegu, Republic of Korea.

^4^Bio-Medical Research Institute, Kyungpook National University, Daegu, Republic of Korea.

*Correspondence:

Sang Jin Lee, MD, PhD

Department of Internal Medicine, Division of Rheumatology

Kyungpook National University School of Medicine, Daegu, Republic of Korea 41944

Tel: 82(053) 200-5492

Fax: 82(053) 426-2046

E-mail: dream1331@knu.ac.kr

**Supplementary table 1: Clinical information of SSc-ILD patients.**

|  | **Case 1** | **Case 2** | **Case 3** | **Case 4** | **Case 5** | **Case 6** |
| --- | --- | --- | --- | --- | --- | --- |
| **General features** |  | | | | | |
| Age | 51 | 66 | 61 | 59 | 60 | 77 |
| Sex | F | F | F | F | F | M |
| GFR (ml/min) | 129.6 | 110.6 | 83.7 | 72 | 77 | 121 |
| ESR (mm/h) | 36 | 28 | 88 | 50 | 46 | 31 |
| CRP (mg/dL) | 0.09 | 0.08 | 0.4 | 0.08 | 0.05 | 0.35 |
| **Autoantibodies**  **(positivity)** |  | | | | | |
| ANA | **+** | **+** | **+** | **+** | **+** | **+** |
| ACAs | **̶** | **̶** | **̶** | **̶** | **̶** | **̶** |
| Anti-scl70 | **+** | **̶** | **̶** | **̶** | **̶** | **+** |
| **Medication (mg)** |  | | | | | |
| Glucocorticoid |  |  |  |  |  | 7.5 |
| MTX | 15 |  | 15 |  |  | 10 |
| MMF |  |  |  | 2000 |  | 2000 |
| Others |  | HCQ  Prograft |  | Prograft |  |  |
| **PFT** |  | N/A |  |  |  | N/A |
| FVC  (%, predicted) | 78 |  | 97 | 72 | 61 |  |
| FEV  (%, predicted) | 86 |  | 103 | 82 | 69 |  |
| DLCO  (mL/mmHg/min) | 70 |  | 75 | 60 | 46 |  |

GFR; glomerular filtration rate, ESR; erythrocyte sedimentation rate, CRP; C-reactive protein, ANA; anti-nuclear antibody, ACAs; anti-centromere antibodies, Anti-scl70; anti-topoisomerase antibody-type of anti-nuclear autoantibodies, MTX; methotrexate, MMF; mycophenolate mofetil, HCQ; hydroxychloroquine, PFT; lung function test, FVC; forced vital capacity, FEV; forced expiratory volume, DLCO; diffusing capacity of the lungs for carbon monoxide. N/A; Non applicable. All patients with SSc included in this table had ILD.

**
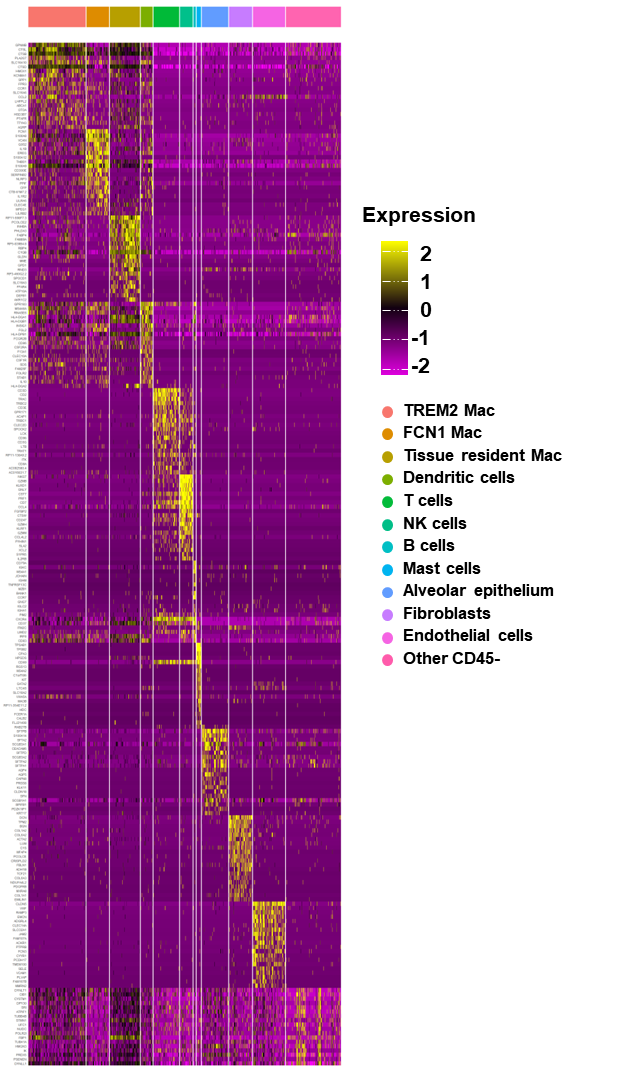
**

**Supplementary Figure 1. Heatmap showing the expression of top 20 differentially expressed genes across the identified cell types.** Gene expression levels are color-coded, with yellow indicating high expression and purple indicating low expression. Mac: Macrophages

**
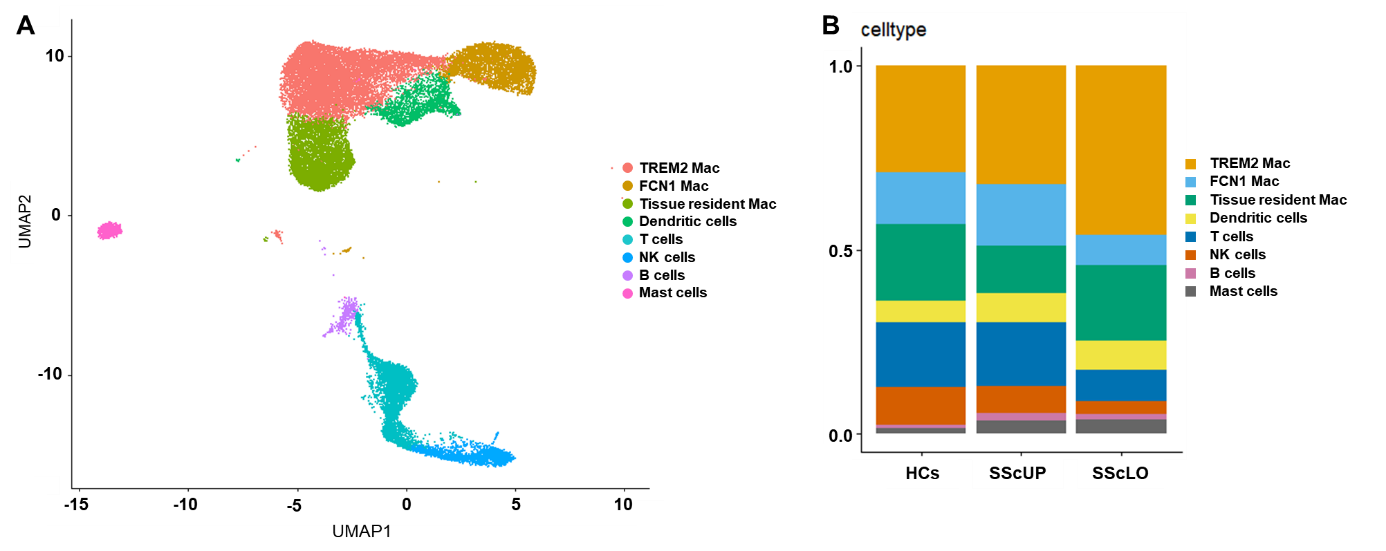
**

**Supplementary Figure 2. CD45⁺ immune-cell subset analysis in lung tissue samples.** (A) UMAP plot of CD45⁺ immune cells subsetted from the whole lung cell population and annotated by major immune-cell populations, including TREM2 macrophages, FCN1 macrophages, tissue-resident macrophages, dendritic cells, T cells, NK cells, B cells, and mast cells. (B) Bar graphs comparing the proportions of immune-cell populations in lung tissues from HCs, SScUP, and SScLO. SScLO was characterized by an increased proportion of TREM2 macrophages and decreased proportions of T cells and NK cells.

**
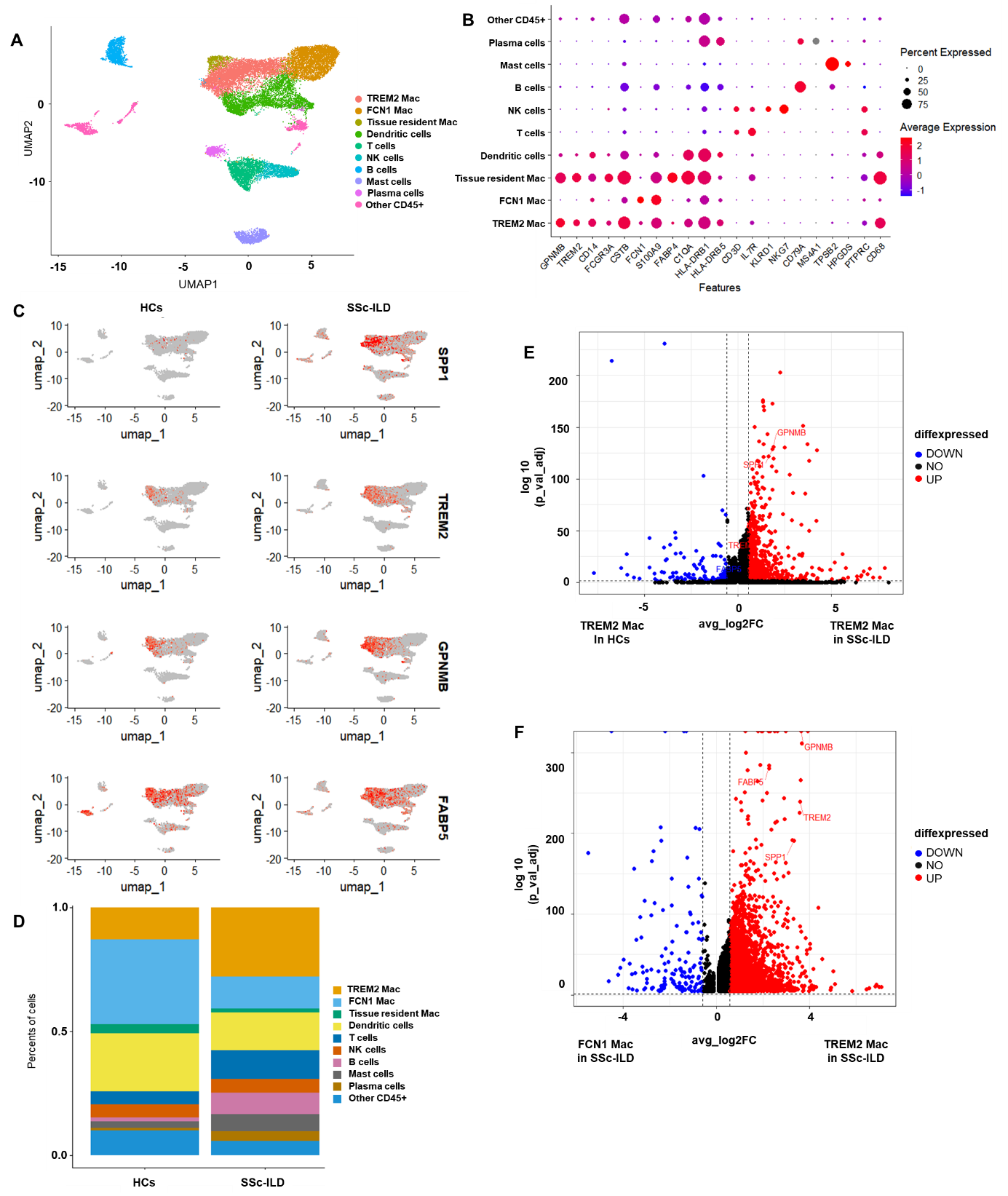
**

**Supplementary Figure 3. Cross-validation of CD45⁺ immune-cell subsets in lung tissues from HCs and SSc-ILD patients using** **an independent publicly available lung scRNA-seq dataset (GSE159354).** (A) UMAP plot of CD45⁺ cells from lung tissue samples, annotated by major immune-cell populations, including TREM2 macrophages, FCN1 macrophages, tissue-resident macrophages, dendritic cells, T cells, NK cells, B cells, mast cells, plasma cells and other CD45⁺ cells. (B) Dot plot displaying representative marker genes for 10 identified cell types. (C) Feature plots comparing the expression of *SPP1*, *TREM2*, *GPNMB* and *FABP5* in lung tissues from HCs and SSc-ILD patients. The intensity of the red color represents the expression level of each gene. Cluster of TREM2 macrophages exhibited the highest expression of SAM-related genes compared to other immune cell types, and were most abundant in the SSc-ILD patients. (D) Bar plot showing the relative proportions of CD45⁺ immune-cell subsets from HCs and SSc-ILD patients. (E) Volcano plot showing significantly upregulated genes (*SPP1*, *TREM2* and *GPNMB*) in TREM2 macrophages from SSc-ILD compared to HCs. (F) Volcano plot comparing TREM2 macrophages with FCN1 macrophages from SSc-ILD, revealing upregulation of *SPP1*, *GPNMB*, *TREM2* and *FABP5*. Genes with significant upregulation are shown in red (average log_2_ fold change > 0.58 and adjusted *p*-value < 0.05), downregulated genes in blue (average log_2_ fold change < −0.58 and adjusted *p*-value < 0.05), and non-significant genes in black. Dotted lines indicate thresholds for statistical significance.

**
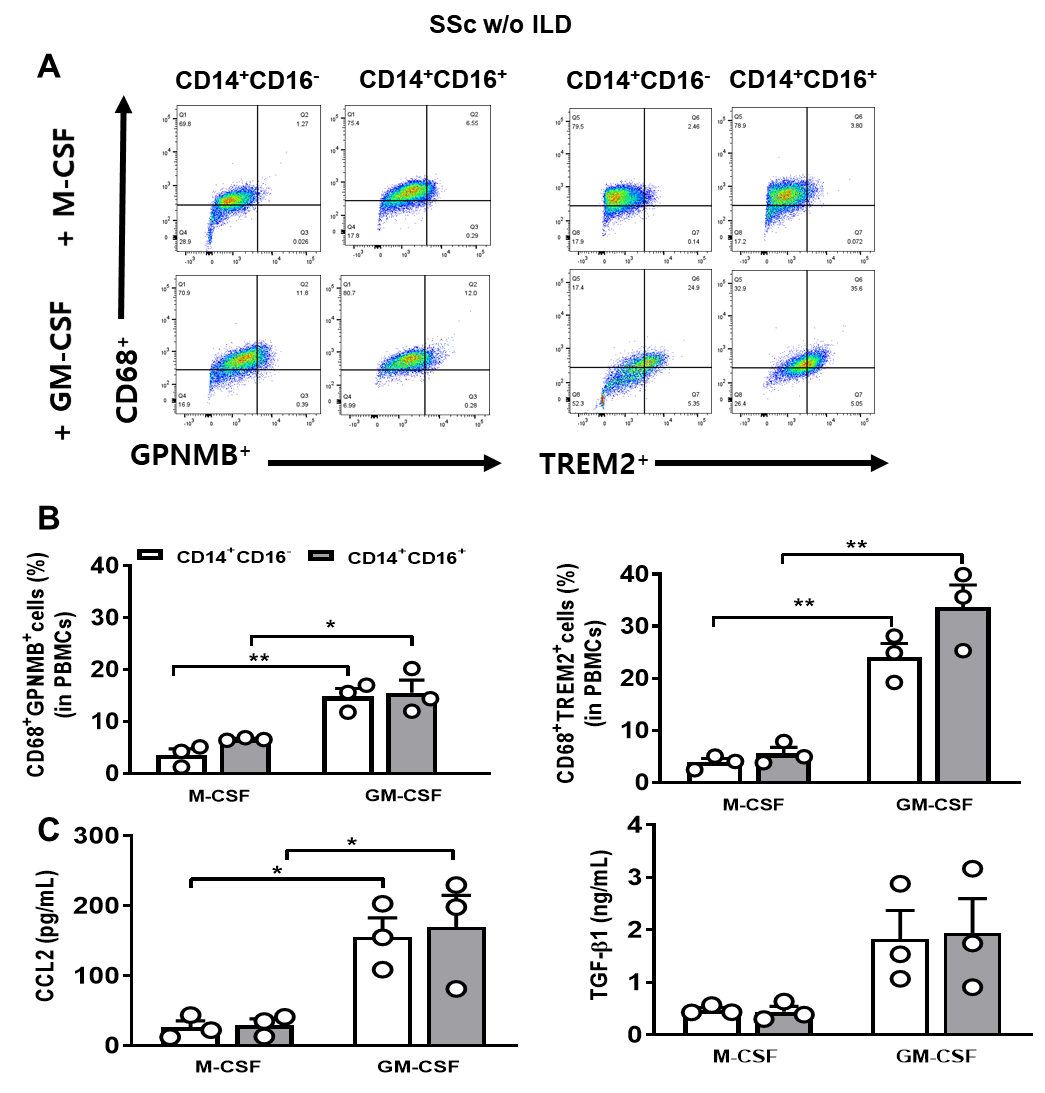
**

**Supplementary Figure 4. Phenotypic and functional characterization of monocyte-derived macrophages from SSc w/o ILD patients.** (A) Representative flow cytometry plots showing GPNMB and TREM2 expression in macrophages differentiated from CD14⁺CD16⁻ or CD14⁺CD16⁺ monocytes of SSc w/o ILD patients cultured with M-CSF or GM-CSF.
(B) Quantification of CD68⁺GPNMB⁺ and CD68⁺TREM2⁺ cells. GM-CSF significantly increased the proportions of CD68⁺GPNMB⁺ and CD68⁺TREM2⁺ macrophages compared with M-CSF. In contrast, no significant differences were observed between CD14⁺CD16⁻- and CD14⁺CD16⁺-derived macrophages under either M-CSF or GM-CSF conditions. (C) Secretion of CCL2 and TGF-β1 levels in culture supernatants. CCL2 levels were significantly higher in GM-CSF–treated cultures than in M-CSF–treated cultures, while no subset-dependent differences (CD14⁺CD16⁻ vs CD14⁺CD16⁺) were detected in either cytokine condition. TGF-β1 levels did not differ significantly between GM-CSF and M-CSF conditions and showed no significant differences between the two monocyte subsets. Statistical analysis was performed using MANOVA, followed by Tukey’s post-hoc multiple-comparisons test. Data are presented as mean ± SEM; n = 3 per group. Statistical significance is indicated as **p* < 0.05 and ***p* < 0.01 for comparisons between M-CSF and GM-CSF within the same subset.


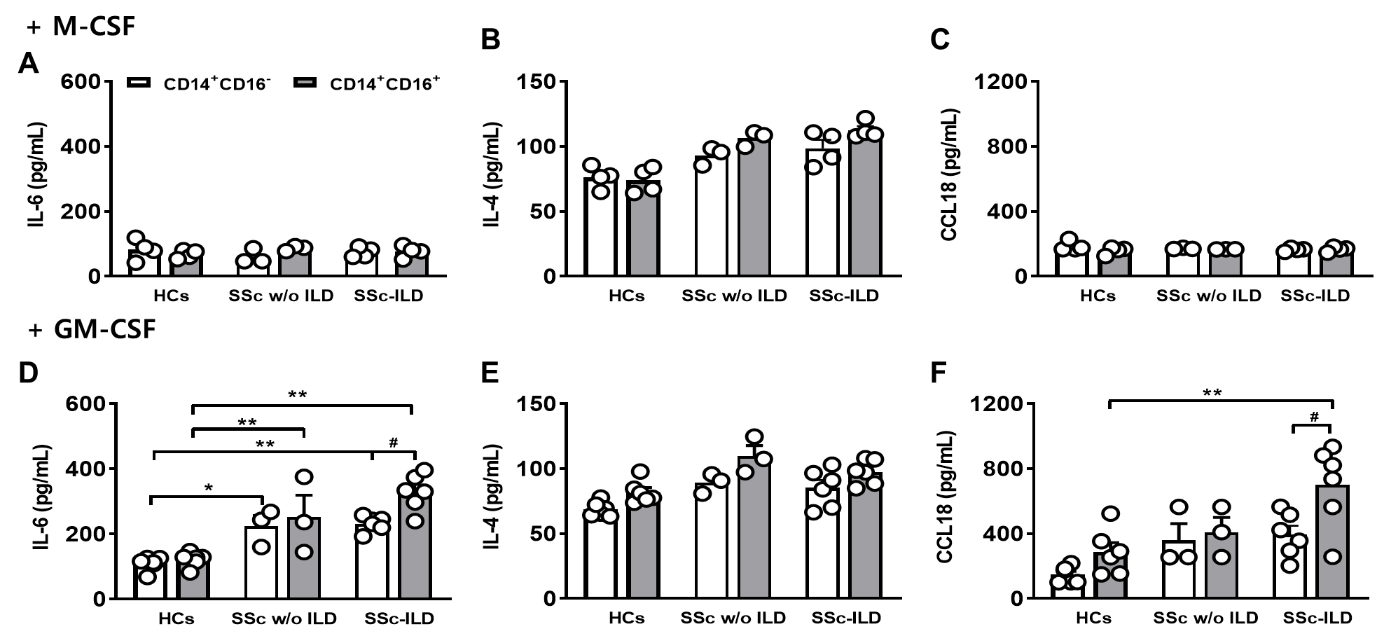


**Supplementary Figure 5. Cytokine and chemokine production by monocyte-derived cells differentiated with M-CSF or GM-CSF.** CD14⁺CD16⁻ and CD14⁺CD16⁺ monocyte subsets were isolated from HCs, SSc w/o ILD, and SSc-ILD patients. Monocytes were differentiated in culturewith M-CSF (A–C) or GM-CSF (D–F). IL-6, IL-4 and CC18 levels were quantified in the culture supernatants. (A) IL-6, (B) IL-4 and (C) CCL18 levels under M-CSF conditions (HCs, n = 4; SSc w/o ILD, n = 3; SSc-ILD, n = 4) showed no statistically significant differences between monocyte subsets (CD14⁺CD16⁻ vs CD14⁺CD16⁺) or among clinical groups (HCs, SSc w/o ILD, and SSc-ILD). (D) IL-6 levels were significantly higher in CD14⁺CD16⁻ derived macrophages in both SSc w/o ILD and SSc-ILD compared with HCs. In the SSc-ILD group, CD14⁺CD16⁺ derived macrophages produced significantly more IL-6 than CD14⁺CD16⁻ derived macrophages. (E) IL-4 levels were comparable across all groups and subsets under GM-CSF conditions. (F) CCL18 levels were increased in the SSc-ILD group compared with HCs, and within SSc-ILD, CD14⁺CD16⁺ derived macrophages produced higher CCL18 than CD14⁺CD16⁻ derived macrophages. Statistical significance is indicated as **p* < 0.05, ***p* < 0.01 for comparisons between each patient group (SSc w/o ILD or SSc-ILD) and HCs within the same monocyte subset; ^#^*p* < 0.05 for comparisons between CD14⁺CD16⁻ and CD14⁺CD16⁺ subsets within SSc-ILD patients.

**
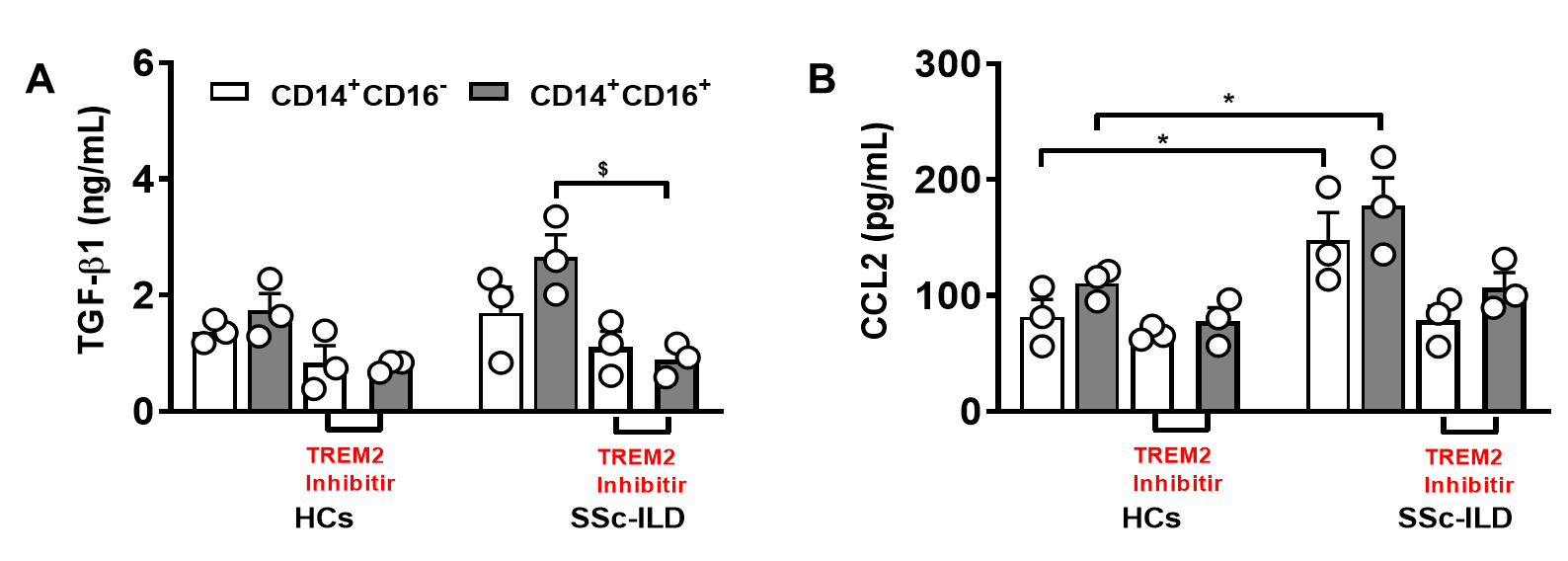
**

**Supplementary Figure 6. TREM2 inhibition attenuates GM-CSF-induced TGF-β1 and CCL2 production.** ELISA was used to measure the levels of (A) TGF-β1 and (B) CCL2 in the supernatants of monocytes derived macrophages co-cultured with lung fibroblasts with or without a TREM2 inhibitor. TREM2 inhibition significantly reduced TGF-β1 secretion in CD14⁺CD16⁺ monocytes derived macrophages from SSc-ILD patients, indicating that TREM2 is required for GM-CSF-induced profibrotic activation. In contrast, although CCL2 levels were elevated in SSc-ILD patients compared with HCs across both CD14⁺CD16⁻ and CD14⁺CD16⁺ macrophage subsets, TREM2 inhibition reduced CCL2 levels, and this decrease did not reach statistical significance. Statistical analysis was conducted using MANOVA, followed by Tukey's post-hoc multiple comparisons test. Data are presented as mean ± SEM, with n = 3 per group. Statistical significance is indicated as **p* < 0.05 between SSc-ILD and HCs within the same subset, ^$^*p* < 0.05 for CD14⁺CD16⁺ monocytes derived macrophages in SSc-ILD vs. those with TREM2 inhibitor.


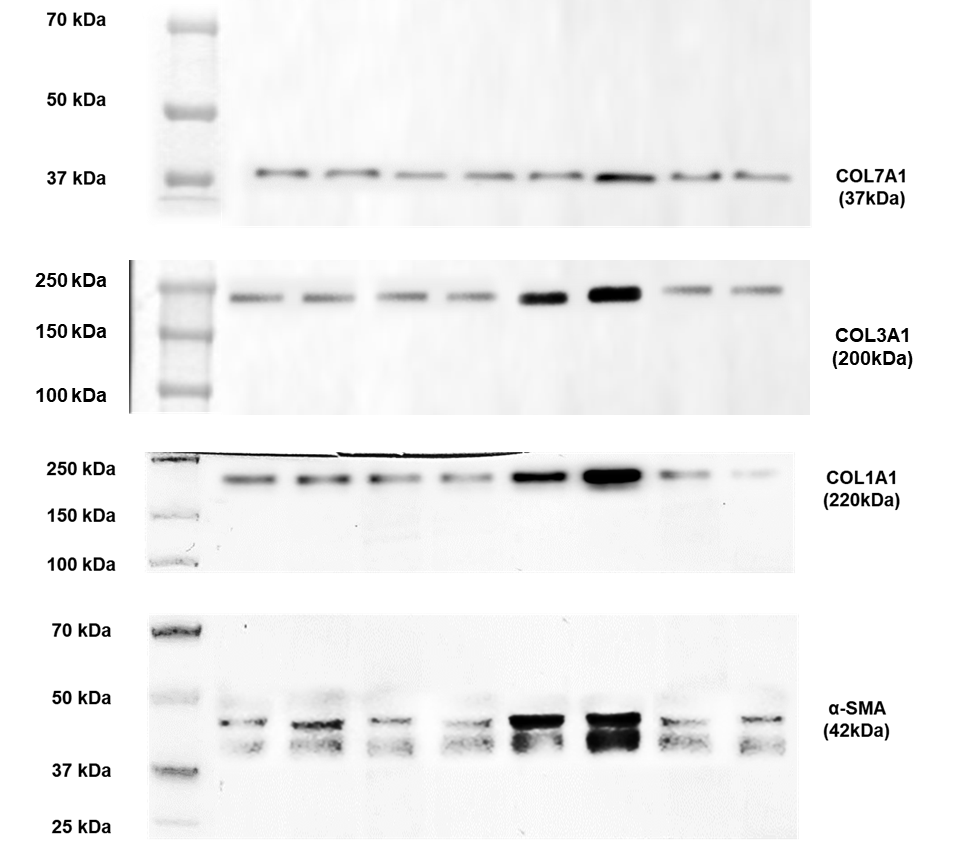


**Supplementary Figure 7. Full-length western blot images for figure 5D.**
